# Characterization of *Heterobasidion occidentale* transcriptomes reveals candidate genes and DNA polymorphisms for virulence variations

**DOI:** 10.1111/1751-7915.13259

**Published:** 2018-04-02

**Authors:** Jun‐Jun Liu, Simon Francis Shamoun, Isabel Leal, Robert Kowbel, Grace Sumampong, Arezoo Zamany

**Affiliations:** ^1^ Natural Resources Canada Canadian Forest Service Pacific Forestry Centre 506 West Burnside Road Victoria BC V8Z 1M5 Canada

## Abstract

Characterization of genes involved in differentiation of pathogen species and isolates with variations of virulence traits provides valuable information to control tree diseases for meeting the challenges of sustainable forest health and phytosanitary trade issues. Lack of genetic knowledge and genomic resources hinders novel gene discovery, molecular mechanism studies and development of diagnostic tools in the management of forest pathogens. Here, we report on transcriptome profiling of *Heterobasidion occidentale* isolates with contrasting virulence levels. Comparative transcriptomic analysis identified orthologous groups exclusive to *H. occidentale* and its isolates, revealing biological processes involved in the differentiation of isolates. Further bioinformatics analyses identified an *H. occidentale* secretome, CYPome and other candidate effectors, from which genes with species‐ and isolate‐specific expression were characterized. A large proportion of differentially expressed genes were revealed to have putative activities as cell wall modification enzymes and transcription factors, suggesting their potential roles in virulence and fungal pathogenesis. Next, large numbers of simple sequence repeats (SSRs) and single nucleotide polymorphisms (SNPs) were detected, including more than 14 000 interisolate non‐synonymous SNPs. These polymorphic loci and species/isolate‐specific genes may contribute to virulence variations and provide ideal DNA markers for development of diagnostic tools and investigation of genetic diversity.

## Introduction

Annosus root and butt rot caused by *Heterobasidion annosum* (Fr.) Bref. *sensu lato* is the most economically important forest disease in the Northern Hemisphere, resulting in serious losses for the forest industry (Woodward *et al*., 1998; Asiegbu *et al*., 2005). Concurrently, wood decay by *Heterobasidion* spp. and related microorganisms is an ecological process essential for nutrient and carbon cycling in the forest ecosystem. Previous morphological and ecological studies classified the *Heterobasidion* species complex into five species. In Eurasia, there are three intersterility groups, *H. abietinum* (F‐type), *H. annosum* sensu stricto (P‐type) and *H. parviporum* (S‐type). In North America, two intersterility groups occur, H*. irregulare* (P‐type) and *H. occidentale* (S‐type). The North American *H. irregulare* has been accidentally introduced into Italy, most likely by the US troops on wood packaging or other military equipment during the World War II (Gonthier *et al*., 2004). The Eurasian and North American intersterility groups (P‐, S‐ and F‐types) were named based on their main host's affinity (pine, spruce or fir) (Capretti *et al*., 1990; Otrosina and Garbelotto, 2010). Although biology, ecology, evolution and management of *Heterobasidion* species have been widely investigated over the past four decades (Otrosina and Garbelotto, 2010), knowledge regarding the pathogen diversity and factors controlling virulence and pathogenicity is still limited for development of effective management strategies to reduce forest resource losses and prevent future damage caused by Annosus root and butt rot particularly under global climate change.

With the development of next‐generation sequencing (NGS), genomes of *H. irregulare* and *H. annosum* were sequenced (Olson *et al*., 2012; Sillo *et al*., 2015; Choi *et al*., 2017). Investigation of the molecular mechanisms underlying fungal penetration and proliferation in the hostile microenvironment of tree tissues implied that enzymatic degradation and efficient efflux mechanisms contribute to pathogenicity, enabling *H. irregulare* to overcome host chemical defence (Olson *et al*., 2012). Comparative genomic study of *H. irregulare* and *H. annosum* genotypes revealed that pathogenicity‐related genes were more conserved between species than genes involved in saprobic growth and sporulation (Sillo *et al*., 2015). As these genome sequences became available, several recent studies reported on more detailed characterization of ABC transporters, CAZyes, cytochrome P450 proteins (CYPome) and candidate effectors in *H. irregulare* (Yakovlev *et al*., 2012; Chen *et al*., 2015; Kuo *et al*., 2015; Lundén *et al*., 2015; Baral *et al*., 2016; Mgbeahuruike *et al*., 2017; Raffaello and Asiegbu, 2017). Linkage map construction, quantitative trait loci (QTL) mapping and genomewide association studies identified genomic regions for *H. annosum* virulence on host conifers (Lind *et al*., 2007, 2012; Dalman *et al*., 2013). Expression analysis identified *Heterobasidion* genes involved in lignin degradation (Yakovlev *et al*., 2013), in response to environmental stressors (Mgbeahuruike *et al*., 2013; Raffaello *et al*., 2014), in association with intersterility (Van der Nest *et al*., 2014) and in interactions with living conifers during early invasion of host tissues (Olson *et al*., 2012; Lundén *et al*., 2015). These efforts provided a much deeper insight into the molecular mechanisms driving compatible interactions in *Heterobasidion* pathosystems.

*Heterobasidion irregulare* and *H. occidentale* are distributed throughout North America, but belong to P‐type and S‐type intersterility groups respectively (Otrosina and Garbelotto, 2010). *H. occidentale* is endemic in western North America (west of the Rocky Mountains) from Alaska to southern Mexico. This fungal pathogen has a wide host range (Garbelotto and Gonthier, 2013). Its major hosts include several forest species with economic and ecological importance: Douglas fir (*Pseudotsuga menziesii*), western hemlock (*Tsuga heterophylla*), various fir (*Abies*) species and Sitka spruce (*Picea sitchensis*). *Heterobasidion irregulare* shares similar morphologies with *H. occidentale* and has capability to infect most of the *H. occidentale* hosts; however, it is mainly found on *Pinus* spp. (P‐type) with distribution largely in North American and Italy's forests. Real‐time PCR assays were used to monitor disease development in host tissues for screening host resistance and to distinguish species in the *H. annosum* species complex (Hietala *et al*., 2003; Lamarche *et al*., 2017). However, few comparative transcriptomic studies have been performed on *Heterobasidion* isolates with a focus on virulence differentiation. *H. occidentale* genetic variability in relation to its pathogenicity and virulence as well as its divergence from other close *Heterobasidion* species (including *H. irregulare*) has not been well characterized. Successful colonization of conifer woody tissues by *H. occidentale* depends on the fungus and host genotypes. Unfortunately, the likelihood of disease emergence cannot be predicted due to a lack of knowledge regarding *H. occidentale* genotypes, in particular those alleles that affect virulent traits.

**Figure 1:**
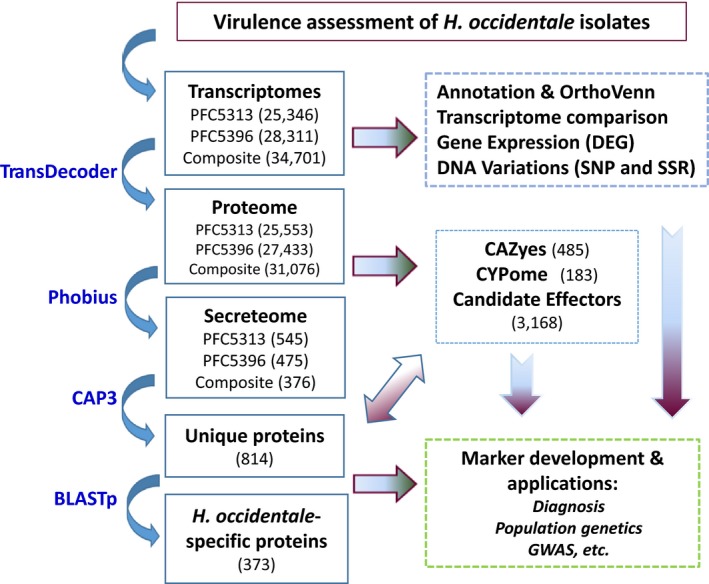
A workflow of an integrated transcriptomic approach for discovery of candidate effectors and DNA markers in *Heterobasidion occidentale* isolates.

In this study, we used an integrated transcriptomics approach (Fig. 1) to identify candidate genes which contribute to virulence variations of *H. occidentale* isolates. Following phenotypic assessment of virulence‐related traits, two isolates with contrasting virulence levels were selected for a comparative global gene expression study using RNA‐seq analysis. Profiling analysis of the secretome, CYPome and other candidate effectors revealed a high degree of core genes conservation and a high degree of diversity of their effectoromes. These data support the hypothesis that the differential expression of a set of lineage‐specific candidate effectors may contribute to differences in virulence among *H. occidentale* isolates. Nucleotide variations, including interisolate non‐synonymous single nucleotide polymorphisms (ns‐SNPs), were further characterized within functional genes. Verification of a subset of molecular markers will facilitate development of a high‐throughput genotyping platform for this wood‐decaying pathogen. As the first comparative transcriptomic investigation of *H. occidentale* isolates, the identification and characterization of genes encoding candidate effectors advance our understanding of the genetic and molecular mechanisms underlying *Heterobasidion* pathogenicity and virulence.

## Results

### *Heterobasidion  occidentale* isolates showed variations of virulence levels

**Figure 2:**
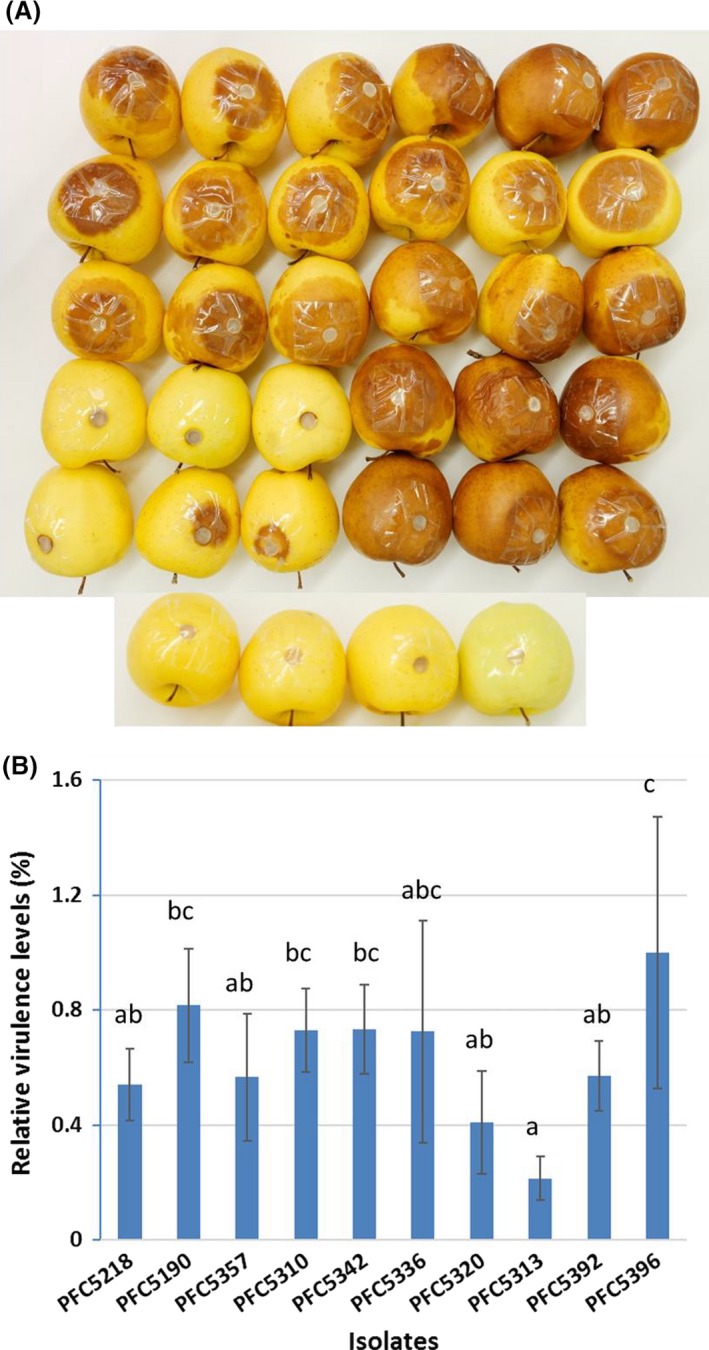
Apple inoculation assay of *Heterobasidion occidentale* isolates. The inoculation experiments were repeated once independently. A. Lesion caused by *H. occidentale* isolates on inoculated ‘Golden Delicious’ apples. From left to right, top to bottom: Row 1: PFC5392 (apples 1‐3), PFC5396 (apples 4‐6); Row 2: PFC5342 (apples 1‐3), PFC 5357 (apples 4‐6); Row 3: PFC5336 (apples 1‐3), PFC5320 (apples‐4‐6); Row 4: PFC5313 (apples 1‐3), PFC5310 (apples 4‐6); Row 5: PFC5218 (apples 1‐3), PFC5190 (apples 4‐6); Row 5: PFC5396 (apples 1–3), PFC5392 (apples 4–6). Row 6: Negative control (apples 1–4). B. Relative virulence analysis of *H. occidentale* isolates. Diseased lesion sizes were measured to calculate relative virulence levels, and different letters indicate a significant difference among isolates (*P* < 0.05 after Bonferroni correction). Error bars show standard deviations.

Ten *H. occidentale* isolates were selected from a wide range of geographical regions (Table S1), and their virulence levels were assessed by an apple inoculation assay. Different virulence levels among isolates were observed following measurement of diseased lesion size post fungal inoculation (Fig. 2A). Relative virulence levels showed significant differences (Fig. 2B). Of ten isolates tested, Hoc‐PFC5396 was the most aggressive with the highest virulence level while Hoc‐PFC5313 was the least aggressive with lesion sizes about five times smaller on average (Bonferroni multiple comparison *P *<* *0.001). The apple inoculation assay was repeated with high correlation between independent experiments (Pearson's correlation coefficient *R* = 0.7586, *P *≤* *0.01), which confirmed Hoc‐PFC5396 and Hoc‐PFC5313 as the most and least aggressive isolates with significant difference. These results demonstrated that the apple inoculation assay was an effective approach to screen for *H. occidentale* virulence. Based on this screening, we selected isolates Hoc‐PFC5313 and Hoc‐PFC5396 for identification of candidate genes involved in fungal virulence and DNA marker development using a transcriptome profiling approach (Fig. 1).

### *De Novo* transcriptome assembly

RNA‐seq was performed on isolates Hoc‐PFC5313 and Hoc‐PFC5396, each with three samples. On average, 38.6 million 100‐bp PE reads were produced per sample with a range from 36.2 to 41.9 million. After filtering out low‐quality reads, a total of 105.2 and 103.0 million clean reads were obtained from isolates Hoc‐PFC5313 and Hoc‐PFC5396 respectively. These clean reads were used for *de novo* assembly of transcriptomes for each individual isolate or in combination as a composite sample (Table 1). Assembly using CLC Genomics Workbench (v5.5) yielded transcriptomes comprised of 25 346 contigs with N50 of 1514‐bp and total length of 22.62‐Mb for isolate Hoc‐PFC5313; 28 311 contigs with N50 of 1250 bp and total length of 22.22 Mb for isolate Hoc‐PFC5396; and 34 701 contigs with N50 of 976 bp and total length of 23.19 Mb for the composite sample (Table 1, Fig. S1A). Assembly quality was checked by read mapping, which revealed that > 96% of total transcripts had at least 10 × average coverage in each of the three assembled transcriptomes (Fig. S1B).

**Table 1:** Summary of *de novo* assemled transcriptomes of *Heterobasidion occidentale* isolates

	PFC5396	PFC5313	Composite sample
De novo transcriptome assemly			
100‐bp PE reads (million)	103	105.2	208.2
N50 (bp)	1,250	1,514	976
Minimum (bp)	201	201	201
Maximum (bp)	15,887	12,777	12,375
Average (bp)	787	888	668
Total transcripts (n)	28,311	25,346	34,701
Total length (Mb)	**22.22**	**22.62**	**23.19**
CDS prediction			
3’‐Prime_partial	5,472	4,781	6,058
5’‐Prime‐partial	7,437	7,223	7,755
Complete	5,483	6,749	4,968
Internal	9,041	6,800	12,295
Total proteins (n)	**27,433**	**25,553**	**31,076**

Coding DNA sequences (CDS) were predicted in the transcriptomes by TransDecoder at cut‐off of 50 codons. A total of 25 553, 27 433 and 31 076 CDS were determined for the transcriptomes of isolates Hoc‐PFC5313, Hoc‐PFC5396 and the composite sample respectively (Table 1). To evaluate the completeness of *H. occidentale* transcriptomes and putative proteomes, reciprocal Blast analysis was performed using *H. irregulare* and *H. annosum* genome sequences or *H. irregulare* proteome. Reciprocal Blast analysis using tBlastn analysis revealed that 89.5–91.3% of putative *H. occidentale* proteomes had significant homology to genome sequences of these two closely related *Heterobasidion* species (*E* value < 1e‐5). Similarly, 86.3% to 88.0% of the *H. irregulare* proteome showed significant homologous hits to the *H. occidentale* transcriptomes (*E* value < 1e‐5) (Table S2). These results suggest that the *H. occidentale* transcriptomes *de novo* assembled from RNA‐seq reads were relatively complete, and species‐unique genes may only account for about 10% of total genes.

**Figure 3:**
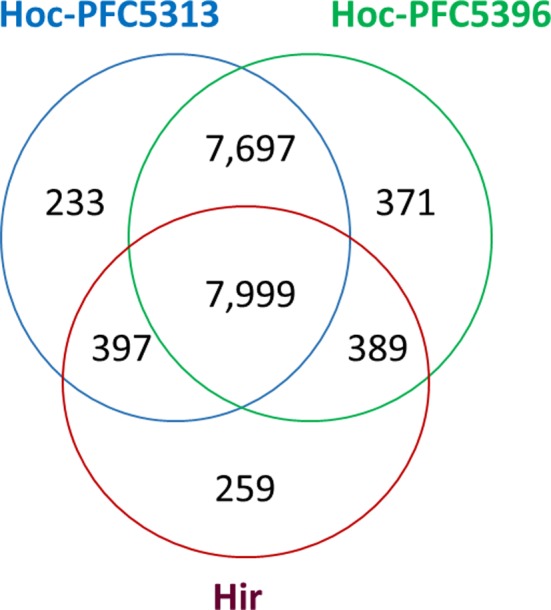
A Venn diagram to show the distribution of orthologous groups (OGs) detected by the OrthoVenn program.

Consistently with the above findings, comparative analysis by OrthoVenn predicted 7999 orthologous groups (OGs) shared by both *H. occidentale* and *H. irregulare*, as well as 233, 371 and 259 OGs unique for the *H. occidentale* isolates Hoc‐PFC5313, Hoc‐PFC5396 and *H. irregulare* isolate TC32‐1 respectively (Fig. 3, Table S3). The hypergeometric tests for gene ontology (GO) enrichment (*P *<* *0.05) found only one biological process (post‐Golgi vesicle‐mediated transport) significantly enriched in isolate Hoc‐PFC5313‐unique OGs. In contrast, isolate Hoc‐PFC5396‐unique OGs were significantly enriched with six GO terms (mannose metabolic process, glucose 6‐phosphate metabolic process, trehalose metabolic process, chaperone‐mediated protein folding requiring cofactor, carbohydrate biosynthetic process and beta‐1,3‐D‐glucan biosynthetic process), suggesting that these biological processes may play roles in *H. occidentale* virulence and pathogenesis.

### Secretome identification and annotation

Fungal secretory proteins have been reported to have different roles in pathogen–host interactions. A total of 814 putative full‐length proteins were determined as secretory proteins due to the presence of N‐terminal single peptides and the absence of TM domains using a bioinformatic pipeline as outlined in Fig. 1. Blastp analysis revealed that 373 of these proteins had no significant homology to the *H. irregulare* proteome (*E* values > e‐5), suggesting that 45.8% of the *H. irregulare* secretome might be species‐specific. These proteins serve as potential candidates as effectors for further studies of fungal adaptation to host conifers.

Only 161 *H. occidentale* secretory proteins were annotated by GO analysis, mainly with putative enzymatic activities for cell wall modification, a process which is crucial for fungal pathogens to penetrate into host plant tissues (Table S4). About 1.6% of the *H. occidentale* proteome, including 90 secretory proteins, were annotated as carbohydrate‐active enzymes (CAZymes) of various families by dbCAN analysis (Table S2, S5). Other annotated secretory proteins had putative functions such as oxidoreductases, proteinases/peptidases, transferases, hydrophobin‐like proteins and isomerases (Table S4).

### CYPome identification, phylogenetic and expression analyses

**Figure 4:**
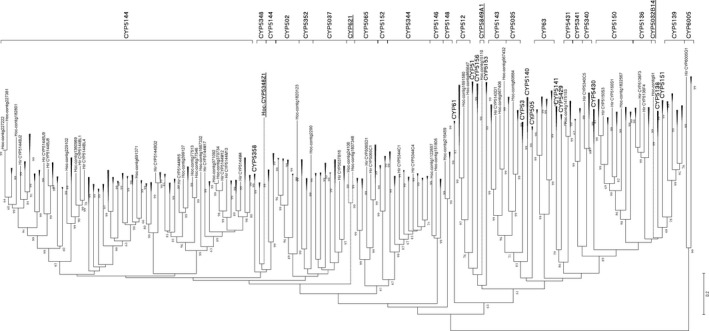
Phylogenetic tree of putative CYP450 proteins from *H. occidentale* (Hoc) and *H. irregulare* (Hir). The 98 pairs of CYP450 proteins clustered well with each other between two species are compressed in black arrows. Three Hoc‐specific CYP families (CYP621, CYP5032 and CYP5849) and CYP5348Z1 are underlined.

A total of 183 unique proteins matching the HMM CYP450 model (PF00067) were mined in the *de novo‐*assembled *H. occidentale* transcriptomes (Fig. 1, Table S6). With CYPome available in *H. irregulare* TC32‐1 isolate (Mgbeahuruike *et al*., 2017), we selected 127 full‐length, or approximately full‐length *H. occidentale* CYP450 proteins for a phylogenetic analysis to address the relationships of this superfamily between two *Heterobasidion* species. Based on alignment analysis of the protein sequences, a phylogenetic tree was constructed, which showing 98 *H. occidentale* CYP450s clustering in pairs with the *H. irregulare* proteins across 34 CYP families (Fig. 4). Although there was a high degree of CYPome conservation between the two *Heterobasidion* species, three additional CYP families (CYP621, CYP5032 and CYP5849) were identified in *H. occidentale*, including CYP5849A1 (Hoc‐contig15110) as annotated for the first time. Using available CYPome databases (Nelson, 2009; Moktali *et al*., 2012) and GenBank nr database, an extensive Blast search found only one other CYP5849 member in *H. annosum* with 95.77% identity to CYP5849A1 (Fig. S2, Moktali *et al*., 2012). Hoc‐contig733973 showed about 40% identity to many fungal CYP5348 proteins, with highest identities of 61.94% and 66.67% to two members in *Hericium erinaceus* (GenBank acc. no. ARE72240) and *Galerina marginata* (GenBank acc. no. KDR74420) respectively (Fig. S3). Hoc‐contig733973 was then annotated as CYP5348Z1, and it showed long divergence from other family members (Fig. 4). Following a Blast search of available genome sequences of four *H. annosum* genotypes and four *H. irregulare* genotypes, CYP5348Z1 was not detected in these two *Heterobasidion* species. Other CYP5032 members were found in the genomes of a few other fungal species and Hoc‐contig91 was clustered with those predicted from genomes of *H. annosum* (Sillo *et al*., 2015) and *Gloeophyllum trabeum* (Floudas *et al*., 2012) (Fig. S4). It is also worth pointing out that two families (CYP512 and CYP5146) were expanded in *H. occidentale*, each with two members more than those in *H. irregulare*. These results demonstrate that *H. occidentale* has gained new CYP families as well as new members of the conserved CYP families since its divergence from *H. irregulare*.

### Other candidate effectors

**Figure 5:**
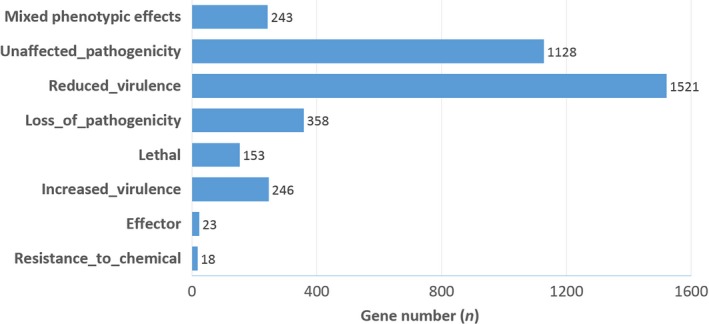
Gene groups with phenotypic effects as predicted by BLAST analysis of *Heterobasidion occidentale* genes against the PHI database.

Other candidate effectors were mined by Blastp search against the PHI database, which revealed that 11.9% of the *H. occidentale* proteome had significant homologous hits (E values cut‐off at e‐5) to the PHI database (Table S2). As expected, 17.9% of the *H. occidentale* secretome and 94.8% of the CYPome were homologous to the PHI proteins (Table S7). These PHI homologs were classified into groups with different phenotypic effects by mutant analysis, including resistance to chemicals, effector, increased or reduced virulence, lethal, loss or unaffected pathogenicity and mixed phenotypic effects (Fig. 5). About half (50.92%) of the PHI homologs were associated with loss of pathogenicity or reduced virulence. It was also noted that 23 *H. occidentale* genes showed significant similarities to those effectors well characterized in other fungal species (Urban *et al*., 2015). All these results demonstrated that a large set of candidate effectors expressed in *H. occidentale* mycelial culture may constitute an effectorome and contribute to phenotypic effects associated with virulence and pathogenicity.

### Genes differentially expressed between isolates

Differentially expressed genes (DEGs) were determined by comparing transcriptomes based on RPKM values. We identified 5118 transcripts, including those encoding 110 secreted proteins, 23 CYP450s and 501 PHI homologs, with fold change > 4 (FDR corrected *P* < 0.01) between two isolates. Furthermore, 1006 and 780 transcripts were found to be expressed exclusively in isolate Hoc‐PFC5313 and Hoc‐PFC5396 respectively. These isolate‐exclusively expressed genes were involved in putative activities related to hydrolases, transferases and oxidoreductases, as well as in binding of nucleic acids, ATP and transition metal ions (Fig. S5).

A few DEGs with nucleic acid binding activities were annotated as transcription factors (TFs) (Table S8). Of four TF families (pathway‐specific nitrogen regulator, transcription factor participating in MBF transcription complex, transcription regulator with C_2_H_2_ Zn finger and transcriptional Swt1), each was detected with two members: one exclusively expressed in Hoc‐PFC5313 and another exclusively expressed in Hoc‐PFC5396. Four contigs encoding proteins with homologies to TF tau subunit sfc1 were detected with one exclusively in Hoc‐PFC5313 and three others exclusively in Hoc‐PFC5396. Furthermore, one transcriptional regulator Prz1 homolog was expressed only in Hoc‐PFC5396 while one homolog of transcriptional activator Acu‐15 was expressed only in Hoc‐PFC5313.

Differentially expressed secretory proteins included glycoside hydrolases of different families, and different members of the aryl‐alcohol oxidase (AAO) and lipase families. As compared to Hoc‐PFC5313, Hoc‐PFC5396 upregulated secretory proteins included glutaredoxin‐related protein, metallopeptidase and serine protease S53, and down‐regulated secretory proteins include carboxylesterase, carbohydrate esterase family 16 protein, GMC oxidoreductase 8, hydrophobin‐like protein, laccase, manganese peroxidase 5 and serine carboxypeptidase S10 (Fig. S6A). Fifteen secretory proteins were exclusively expressed in either Hoc‐PFC5313 or Hoc‐PFC5396 but were not annotated.

Of 37 *H. occidentale* CYP families, 12 families with 23 members were differentially expressed between two isolates (Fig. S6B). In particular, CYP5849A1 (contig15110) was expressed exclusively in isolate Hoc‐PFC5396, not in Hoc‐PFC5313. Similarly, CYP5849A1 ortholog also showed to be isolate‐specific among four *H. annosum* genomes by Blast analysis, with presence of a complete CDS in Han‐03012 (FBL) and Han‐137OC, but was absent in two other isolates (Han‐BM42NG and Han‐109SA) due to an incomplete CDS.

### *In silico* identification of nucleotide variations

**Figure 6:**
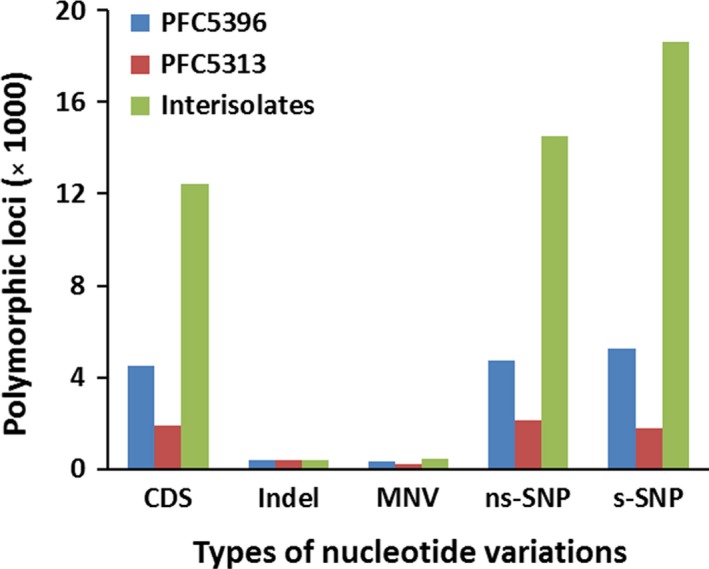
Characterization of nucleotide variations in *Heterobasidion occidentale* isolates. Protein‐coding regions were predicted by TransDecoder and used as reference sequences for mapping RNA‐seq reads in detection of nucleotide variations.

Intra‐isolate nucleotide variations were first detected. Of all transcripts, 1919 and 4521 transcripts were detected with 4477 and 10 714 polymorphic loci in Hoc‐PFC5313 and Hoc‐PFC5396 respectively. Of these intra‐isolate polymorphic sequences, only 651 were polymorphic in both isolates. As expected, most of the nucleotide variations were SNPs, which accounted for 87.1% and 93.1% of total polymorphic loci in the two isolates, while insertion/deletion (indel) and multinucleotide polymorphism (MNP) accounted for only small proportions of the total (Fig. 6). Hoc‐PFC5396 displayed twice the amount of SNPs per Kb transcript than Hoc‐PFC5313 (0.63 vs. 0.28). In contrast to low intra‐isolate variations, a large number of nucleotide variations were detected between the two isolates. A total of 12 432 CDS contained interisolate polymorphic loci, including 475 indels, 602 MNVs and 33 168 SNPs. About 43.8% of SNPs were non‐synonymous (ns‐SNP), causing amino acid changes (Fig. 6).

**Figure 7:**
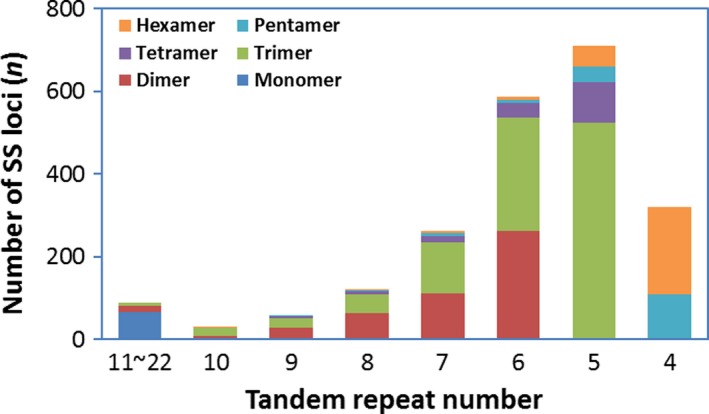
Types and tandem repeat numbers of SSR loci *in silico* mined in the *Heterobasidion occidentale* transcriptome.

We detected a total of 2177 *in silico* SSR loci within 1788 unigenes (Table S9), which accounted for about 5% of the total transcripts. Of these unigenes with SSR distribution, 106 unigenes contained more than one EST‐SSR motifs. On average, one EST‐SSR locus per 10‐Kb sequence was counted across the transcriptome. The most abundant type of repeats were trimer repeats (46.81%), followed by dimer (22.23%), hexamer (12.91%), pentamer (7.53%), tetramer (7.44%) and monomer (3.08%) repeats (Fig. 7). A total of 424 distinct SSR motifs were identified with 2, 8, 28, 60, 113 and 213 unique SSR motifs for mono‐, di‐, tri‐, tetra‐, penta‐ and hexamer repeats respectively. The dimer repeats GC/GC and CG/CG were the most dominant SSR motifs, which accounted for 5.60% and 4.96% of total SSR loci, followed by three types of trimer repeats CGA/TCG, CAG/CTG and GCA/TGC, each accounting for 4.27% of total SSR loci. For all SSR loci, the tandem repeats ranged from four to 22 times, with five tandem repeats being most frequent (32.66% of the total), followed by six (26.96%), four (14.75%) and seven tandem repeats (12.03%). Of all EST‐SSR loci, 1,683 primer pairs were designed by the Primer3 program, which covered 1493 unigenes (Table S10).

### Phylogenomic relationship among *Heterobasidion* spp.

**Figure 8:**
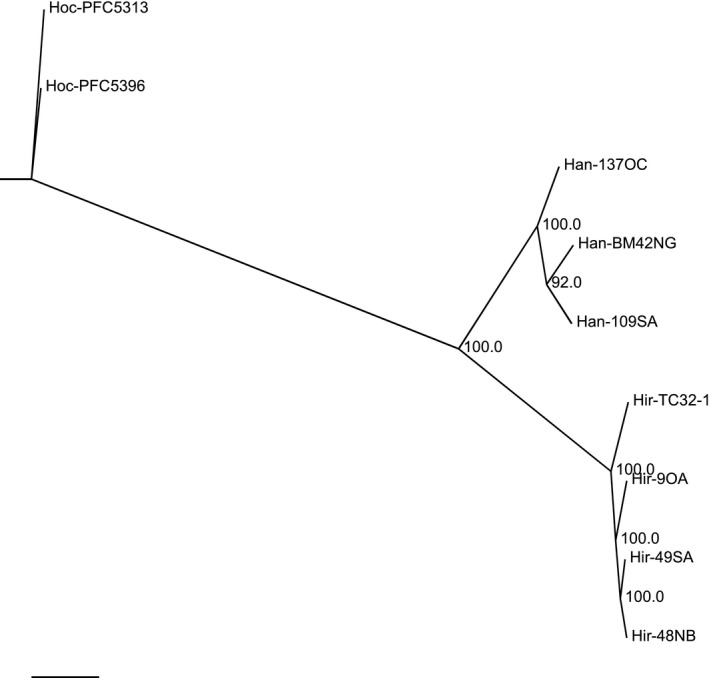
Phylogenetic tree showing the phylogenomic relationship among different isolates of *Heterobasidion occidentale* (Hoc), *H. irregulare* (Hir) and *H. annosum* (Han).

Whole‐genome coding sequences of *H. irregulare* TC32‐1 were used as a reference for phylogenomic analysis. A consensus phylogenetic tree was constructed among nine genotypes/isolates of three *Heterobasidion* species. As shown in Fig. 8, genotypes/isolates of the same species were closely clustered together, clearly demonstrating two distinct *Heterobasidion* species in North America.

## Discussion

In this study, we used a genomic approach to identify candidate genes contributing to *H. occidentale* infection and disease development. First, an apple inoculation assay was used to evaluate virulence‐related traits (De Lange *et al*., 1998; Plourde and Bernier, 2014). This method offers us a quick and easy way to asses*s Heterobasidion* virulence levels, which in turn may speed up profiling genetic differences of a large collection of isolates/genotypes. A preliminary screening of 10 representative *H. occidentale* isolates identified two isolates with contrasting virulence level, one highly virulent (Hoc‐PFC5396) and one least virulent (Hoc‐PFC5313). Based on virulence screening results, transcriptomes of these two isolates with contrasting virulence levels were profiled by *de novo* assembly of RNA‐seq reads, leading to identification of OGs that are unique to *H. occidentale* and its isolates by comparative analysis of the putative proteomes. Thirdly, secretome, CYPome, and effectorome, as well as their potential up‐stream regulators, were characterized to aid in a comprehensive understanding of the genetic components contributing to virulence at the transcriptome level. Finally, genetic variations within the transcriptomes were determined for application in future studies. All of these outcomes improve the genomic resources necessary for the detection and evaluation *of H. occidentale* genetic diversity, leading to the final development of a sustainable management strategy for the prevention and suppression of Annosus root disease in western North America.

A network of genes involved in pathogenicity and virulence was uncovered by a comparative transcriptomic study. We found that TFs from multiple families and discovered a large proportion of secretome, CYPome and other candidate effectors, which were differentially expressed between isolates with contrasting virulence levels. Differentially expressed TFs included Prz1, Acu‐15, Swt1 and others, suggesting that each TF may control a set of its targeted genes in signalling networks in *H. occidentale*. Transcriptional activator Acu‐15 positively regulates expression of specific genes involved in the fungal metabolism of other carbon sources (Bibbins *et al*., 2002). A *Histoplasma capsulatum* Acu‐15 ortholog catalyses both cell shape change and the increased expression of known virulence factors, thus functioning as a molecular linker between temperature and the expression of traits required to cause disease in the host (Beyhan *et al*., 2013). Prz1 regulates calcium ion homeostasis by binding to the calcineurin‐ or Ca^2+^‐dependent response element in the promoters of its targeted genes (Hirayama *et al*., 2003). Fission yeast Prz1 protein positively and negatively regulates hundreds of targeted genes, which are involved in biological processes such as cell wall biosynthesis and others through a calcineurin‐mediated transcriptional regulatory network (Chatfield‐Reed *et al*., 2016). Yeast Swt1 probably functions as a transcriptional activator, its deletion causes a slow growth phenotype and leads to decreased transcription (Roether *et al*., 2006). Further studies are required to determine if *H. occidentale* homologs of Prz1, Acu‐15, Swt1, or other TFs cause differential expression of candidate effectors and CYPome between isolates, thus contributing to virulent traits.

CYP450s are haem‐thiolate proteins universal in all biological kingdoms. A surprisingly large number are present in fungal genomes, especially in wood‐degrading basidiomycetes (Park *et al*., 2008; Črešnar and Petrič, 2011; Nelson, 2011; Chen *et al*., 2014a). The evidence accumulated so far demonstrates that CYP450s make significant contribution to the fitness and fecundity of fungi in various ecological niches through their catalytic versatility, which impacts the metabolism of a diverse array of endogenous and xenobiotic compounds (Syed and Mashele, 2014). Our study identified the *H. occidentale* CYPome by mining *de novo*‐assembled transcriptomes to characterize species‐ and isolate‐exclusive members. P450s were found with significant regulation during pathogen–host interactions in a number of fungal pathogens (Siewers *et al*., 2005; Karlsson *et al*., 2008; Leal *et al*., 2010; Zhang *et al*., 2012; Gao *et al*., 2014). CYP450 participated in mycotoxin synthesis, playing an essential role in the pathogenic process in several fungi (Roze *et al*., 2013). Compared to the *H*. *irregulare* CYPome (Mgbeahuruike *et al*., 2017), higher gene diversity level was revealed in *H. occidentale* as evidenced by greater family number and more member numbers of families. This probably implies a diversification of enzymatic activities among *H. occidentale* isolates to meet diverse metabolic demands for adaptations to their ecological niches. CYP5849 was a novel family annotated in the fungal CYPomes. CYPs have shown potential as diagnostic tools for identification and differentiation of fungal species (Okhravi *et al*., 1998). CYP5348Z1 was characterized as unique to *H. occidentale* and absent in the other two genome‐sequenced *Heterobasidion* species, indicating its potential application in identification of *H. occidentale* in field samples.

CYP5849A1 was detected in the more virulent Hoc‐PFC5396, but was absent in Hoc‐PFC5313 with the lowest virulence level. The CYP5849 family was not identified in other wood‐decaying fungi with sequenced genomes with the exception of the two genome‐sequenced *H. annosum* isolates, suggesting that it may possess a distinct role in the production of divergent metabolites critical for isolate‐specific pathogenesis. VdCYP1 is one necessary pathogenic factor during infection of cotton by *Verticillium dahliae*, which may be involved in the synthesis of secondary metabolites (Zhang *et al*., 2016). Future studies will be needed to elucidate the pathogenic role CYP5849A1 (contig15110) may have.

Coupled with the three additional CYP families identified in the *H. occidentale* transcriptome, families CYP512 and CYP5146 were expanded in *H. occidentale* as compared to *H. irregulare*. A total of 23 members of 12 CYP families were found to be differentially expressed between isolates. Some of these families (CYP512, CYP5035, CYP5037 and CYP5136) have also been reported to be enriched in wood‐decaying fungi, suggesting a role in the oxidation of plant materials for fungal adaptation to diverse ecological niches (Hirosue *et al*., 2011; Ide *et al*., 2012). There is evidence that CYP450s in pathogenic fungi are able to overcome plant chemical defence. For example, members of enriched families CYP512, CYP5035, CYP5037 and CYP5136 are capable of detoxifying and degrading defence‐related chemicals (such as resins and coumarin) by their oxidation activity, playing a crucial role in fungal colonization inside wood tissue (Syed and Mashele, 2014). These species‐, isolate‐specific, or expanded CYP families provide compelling candidates for further studies of their substrate specificity to better understand *H. occidentale's* adaptation to specific conifer hosts.

Effectors are mostly secretory proteins and act as key governing proteins that manipulate host defence mechanisms and result in either an incompatible (resistant) or a compatible (susceptible) interaction, depending on host genotypes. Candidate effectors usually share some common characteristics, including presence of a signal peptide for secretion, absence of both transmembrane domains, a lack of PFAM domains and fairly small size, and are mostly species‐specific (Sonah *et al*., 2016). These features are widely used for bioinformatic predictions of candidate effectors in numerous fungal species based on genome or transcription sequences (Liu *et al*., 2015; Sonah *et al*., 2016; Raffaello and Asiegbu, 2017), which provides promising candidates for further experimental analyses to elucidate molecular interactions and co‐evolution of hosts and pathogens. This study identified 814 putative secretory proteins in *H. occidentale* using Phebious, only 20% of which were annotated with enzymatic activities (such as CAZymes), mainly involved in cell wall modification. Other secretory proteins were revealed as genus‐, species‐ and even isolate‐specific without any homologous hits to the available secretome databases, including that of *H. irregulare*. The gene expression study further found members of GH12, GH23, GT69, AAO, MPsae associated with high virulence level. Consistently, carbohydrate biosynthetic process and beta‐1,3‐D‐glucan biosynthetic process were two GO terms enriched for the OGs specific to the virulent isolate (Hoc‐PFC5396). AAOs are important secreted flavoproteins with an essential role in natural lignin degradation (Hernandez‐Ortega *et al*., 2012), and they produce H_2_O_2_ as a continuous source of cooxidant for peroxidases to degrade lignin during the fungi's attack to the plant cell wall (Ruiz‐Dueñas and Martinez, 2009). The family M35 Zn^2+^ metallopeptidase is important in the virulence of *Aeromonas salmonicida*, playing a major role in the host innate immune response (Arnadottir *et al*., 2008). The *H. occidentale* candidate effectors, in particular those with isolate‐exclusive expression without any annotated function, will be selected for functional analysis and diagnostic tool development in future studies.

The large numbers of SNP and SSR loci reported in the present study have potential applications in future population genetics and genome wide association (GWA) studies, enhancing current knowledge and genomic resources that are pivotal for the development of management strategies for long‐term control of forest pathogens. Such genomics‐based approaches have demonstrated their usefulness in the management of fungal pathogens. SNP‐based GWA identified genomic regions for virulence in *H. annosum* (Dalman *et al*., 2013; Bartoli and Roux, 2017). SNPs and SSRs have been applied in detection, identification and traceability of fungal pathogens (Canfora *et al*., 2016), including the *Heterobasidion* species complex (Lamarche *et al*., 2017). Availability of *H. occidentale* SNP and SSR databases will facilitate diagnosis and monitoring virulent isolates across the landscape of this important forest pathogen.

In conclusion, the transcriptome is first reported for isolates of *H. occidentale*, a forest pathogen for serval important conifers in western North America. The genomic knowledge obtained here improves our understanding of virulent and pathogenicity‐related traits in the *Heterobasidion* species complex. Application of genomics resources (such as SSR and SNP DNA markers) in population genetics and GWA studies is required for the development of genomics‐based strategies for effective management of Annosus root and butt rot disease.

## Experimental procedure

### Fungal isolates and inoculation

Ten *Heterobasidion occidentale* (Hoc) isolates (Table S1) were cultured on 2% malt extract agar (MEA; DIFCO) at room temperature (~20°C). We tested their relative virulence levels using an apple inoculation assay (De Lange *et al*., 1998; Plourde and Bernier, 2014). The surfaces of apple fruits (Golden Delicious) were washed and sterilized using 70% ethanol and then rinsed with sterilized water. A hole about 1.5 cm deep was cut in each apple fruit using a 9‐mm core borer. An inoculum plug taken at growth phase was inserted into the hole with the mycelial side facing inwards. The inoculation sites were covered by transparent packing tape to prevent outside contamination and desiccation. The apples were placed in Tupperware containers in the incubator at 25°C in the dark. Lesion diameters were measured at different days post‐inoculation (dpi). At least eight apples were used in inoculation test for each isolate and a sterile MEA plus served as negative control. The apple inoculation assay was repeated independently once in a different year. One‐way ANOVA with Bonferroni correction was used to estimate statistical significance for differences among isolates.

### RNA‐seq analysis and *de novo* assembly of transcriptome

For RNA extraction, a plug of 7‐day‐old actively growing isolate on an MEA plate was transferred into 2% malt extract broth and cultured with 25 ml broth in 50‐ml Falcon tubes for 7 days. Mycelium was harvested by spinning down at 10 000 rpm. The wet mycelia pellet was transferred into a tube with lysing matrix A (MP Biomedicals, Solon, OH, USA) and ground into fine powder under frozen conditions using liquid nitrogen. Total RNA was extracted using RNeasy plant mini kits (Qiagen, Toronto, ON, Canada). Genomic DNA was digested with RNAse‐free DNAse (Ambion Inc, Austin, TX, USA) at 37°C for 45 min, and then, the DNAse was deactivated and removed using the Qiagen RNeasy spin column. Clean RNAs were finally eluted using nuclease‐free water in a volume of 100 μl.

RNA‐seq analysis was performed as described previously (Liu *et al*., 2015). The Illumina HiSeq‐2000 platform was used to generate 100‐bp paired‐end (PE) reads at Génome Québec Innovation Centre, McGill University. Raw reads were first trimmed using Trimmomatic software (Bolger *et al*., 2014), with default settings. Clean PE reads from each isolate were combined for *de novo* assembly using CLC Genomics Workbench v5.5 (CLC Bio, Cambridge, MA, USA) with graph parameters of automatic word size and automatic bubble size. The parameters for mapping read back to the contigs were set at mismatch cost = 2, length fraction = 0.95, similarity fraction = 0.95, deletion or insertion cost = 3 and minimum contig length = 201. The *H. occidentale* transcriptome assemblies and RNA‐seq reads have been deposited at GenBank under BioProject ID: PRJNA429253.

### Annotation of candidate effectors and other proteins involved in pathogenicity

Coding DNA sequences (CDS) were identified by TransDecoder (http://transdecoder. sourceforge.net/) with a minimum open reading frame (ORF) size of 50 codons. The predicted proteome was annotated by similarity searches in the NCBI (National Center for Biotechnology Information) nonredundant protein sequence (NR) database using the Blast2Go program (Conesa *et al*., 2005). For comparative analysis, the *H. irregulare* TC 32‐1 genome and proteome sequences were downloaded from the JGI database (Olson *et al*., 2012) and *H. annosum* genome sequences were downloaded from GenBank (Sillo *et al*., 2015). BLAST analysis was performed to compare *H. occidentale* sequences with *H. irregulare* and *H. annosum* genome sequences or *H. irregulare* proteome (13 275 proteins). Orthovenn server (Wang *et al*., 2015) was used to determine orthologous gene families.

Secretome was identified by scanning complete ORFs using Phobius (Käll *et al*., 2007), which generated a list of high‐confidence effectors with the presence of signal peptide and absence of any transmembrane domain. The *H. occidentale* secretome was compared with the *H. irregulare* proteome predicted from the *H. irregulare* TC32‐1 reference genome by Blast analysis. In addition to the secretome identified in full‐length ORFs, other candidate effectors were revealed by Blast search against the PHI (pathogen–host interactions) database, which contained 4003 proteins (Urban *et al*., 2015). The dbCAN web server (HMMs 6.0) was used to identify putative CAZymes by searching against CAZyDB with default parameters (released 07/20/2017) (Yin *et al*., 2012).

To identify CYPome, *H. occidentale* proteins were subjected to HMMSCAN using profile‐HMM database with an *E*‐value cut‐off of 1e‐5 (http://www.ebi.ac.uk/Tools/hmmer/search/hmmscan). Then, sequences matching the HMM model (PF00067) with positive hits were analysed in an annotation procedure by Blast comparisons against Dr. Nelson's cytochrome P450 database (http://drnelson.uthsc.edu/fungal.genomes.html; Nelson, 2009) and the Fungal Cytochrome P450 Database (http://p450.riceblast.snu.ac.kr/species.php, Moktali *et al*., 2012), as well as the CYPome (121 proteins assigned to 34 families) in the *H. irregulare* TC32‐1 isolate (Mgbeahuruike *et al*., 2017) and 135 putative P450 proteins without family assignment in the *H. annosum* FBL isolate (Moktali *et al*., 2012). The full‐length proteins were identified based on previously reported criteria (Chen *et al*., 2014b). These predicted CYPs were assigned to corresponding family types based on their highest sequence similarity (at least 40%) against annotated fungal CYPs.

### Detection of nucleotide variations

Because single nucleotide polymorphism (SNP) loci within coding regions may be non‐synonymous substitutions with effects on protein functions, we focused on *in silico* detection of nucleotide variations in the coding regions of the transcriptome. The coding sequences were used as reference for detection of nucleotide variations within and between isolates. Clean RNA‐seq reads were mapped with parameters as described above. Quality‐based variant detection was performed using CLC program with parameters: neighbourhood radius at 5, maximum gap and mismatch count at 2, minimum neighbourhood quality at 15, minimum central quality at 20 and minimum variant frequency at 35%.

The transcriptome sequences were searched for SSR loci with motifs of one to six nucleotides in size using the SSR Locator program (da Maia *et al*., 2008). The minimum repeat numbers were defined as 12, 6, 5, 5, 4 and 4 for the monomer to hexamer motifs respectively. Primer pairs from the flanking sequences of identified SSRs were designed using Primer3 program (Rozen and Skaletsky, 2000).

### Gene expression analysis

Illumina RNA‐seq reads were mapped back to the reference transcriptome to evaluate transcript expression levels using CLC Genomics Workbench with parameters: minimum length of putative exons = 50, minimum number of reads = 10, maximum number of mismatches = 2, unspecific match limit = 10, minimum exon coverage fraction = 0.2, minimum length fraction = 0.95 and minimum similarity fraction = 0.95. Paired reads per kilobase of transcript per million mapped reads (RPKM) were used to calculate transcript expression values. RPKM values were normalized with parameters: normalization method = scaling, scaling method = mean, trimming = 5% and normalized data = original expression values. The Student's *t*‐test was used to estimate statistical significance of differences of transcript levels between isolates. Genes with normalized fold change > 4 at *P* < 0.01 after adjustment for false discovery rate (FDR) were called to detect differentially expressed genes (DEGs). The FDR was controlled by the Benjamini–Hochberg method (Benjamini and Hochberg, 1995).

### Phylogenomic and phylogenetic analyses

The phylogenomic relationship among *Heterobasidion* species and isolates/genotypes was determined by phylogenomic analysis using REALPHY (Bertels *et al*., 2014). Raw reads of two *H. occidentale* isolates, and six genotypes of *H. irregulare* and *H. annosum* (Sillo *et al*., 2015), were included for read mapping to a reference that was the coding sequences of the *H. irregulare*‐TC32‐1 proteome (Olson *et al*., 2012). As integrated in REALPHY, Bowtie2 (Langmead *et al*., 2009) was used for read mapping; and PhyML (Guindon *et al*., 2010) was used for construction of a phylogenetic tree using the maximum‐likelihood method by bootstrapping with 1000 repeats.

The phylogenetic relationships between the CYPomes of *H. occidentale* and *H. irregulare* were analysed using MEGA5.0 (Tamura *et al*., 2011). Protein sequences of *H. irregulare* CYPome (Mgbeahuruike *et al*., 2017) were aligned with those identified in *H. occidentale* by the MUSCLE algorithm (Edgar, 2004) for multiple sequence alignment with high accuracy and high throughput using the EMBL‐EBI on‐line server. The phylogenetic tree was constructed based on aligned sequences determined by the neighbour‐joining (NJ) method by p‐distance in MEGA5. The significance level for NJ analysis was examined using 1000 bootstrap replicates.

## Conflict of interest

None declared.

## Supporting information


**Fig. S1.** Transcriptomes *de novo* assembled by RNA‐seq analysis.

**Fig. S2.** Protein sequence alignment of *H. occidentale* CYP5849A1 (Hoc‐contig15110) with its *H. annosum* ortholog (Han‐03520).

**Fig. S3.** Protein sequence alignment of *H. occidentale* CYP5348Z1 (Hoc‐contig733973) with its putative orthologs from *Galerina marginata* (KDR74420) and *Hericium erinaceus* (ARE72240).

**Fig. S4.** Phylogenetic analysis of fungal CYP5032 family.

**Fig. S5.** GO terms of *H. occidentale* genes with isolate‐exclusive expression patterns.

**Fig. S6.** Heat maps showing the expression pattern of differentially expressed genes (DEGs) between the *H. occidentale* two isolates.

**Table S1**. *Herobasidion occidentale* isolates invetigested in the present study.
**Table S2.** Blast analysis of *Heterobasidion occidentale* transcriptome *de novo* assembled by RNA‐seq.
**Table S3**. Prediction of orthologous clusters by the OrthoVenn program.
**Table S4**. Heterobasidion occidental secreted proteins as annotated by BlastP analysis using *H. irregulre* proteome.
**Table S5**. A list of 90 sectreted proteins identified as CAZymes by dbCAN annotation.
**Table S6**. *Heterobasidion occidentale* CYP450 protein sequences and their holomolgies to the *H. irregulre* CYProme.
**Table S7**. Blastp analysis of *H. occidentale* secretome and CYProme using PHI database.
**Table S8**: Putative transcription factors with isolate‐exclusive expression pattern.
**Table S9**. Summary of the EST‐SSRs that were identified in the *Heterobasidion occidentale* transcriptome.
**Table S10**. PCR primers for amplication of SSR loci.
