# Supplementary figures and images for: Characterization of Heterobasidion occidentale transcriptomes reveals candidate genes and DNA polymorphisms for virulence variations
Source: Microb Biotechnol. 2018 Apr 2;11(3):537–50. doi: 10.1111/1751-7915.13259 (PMC5954486; doi:10.1111/1751-7915.13259)

## Slide 1
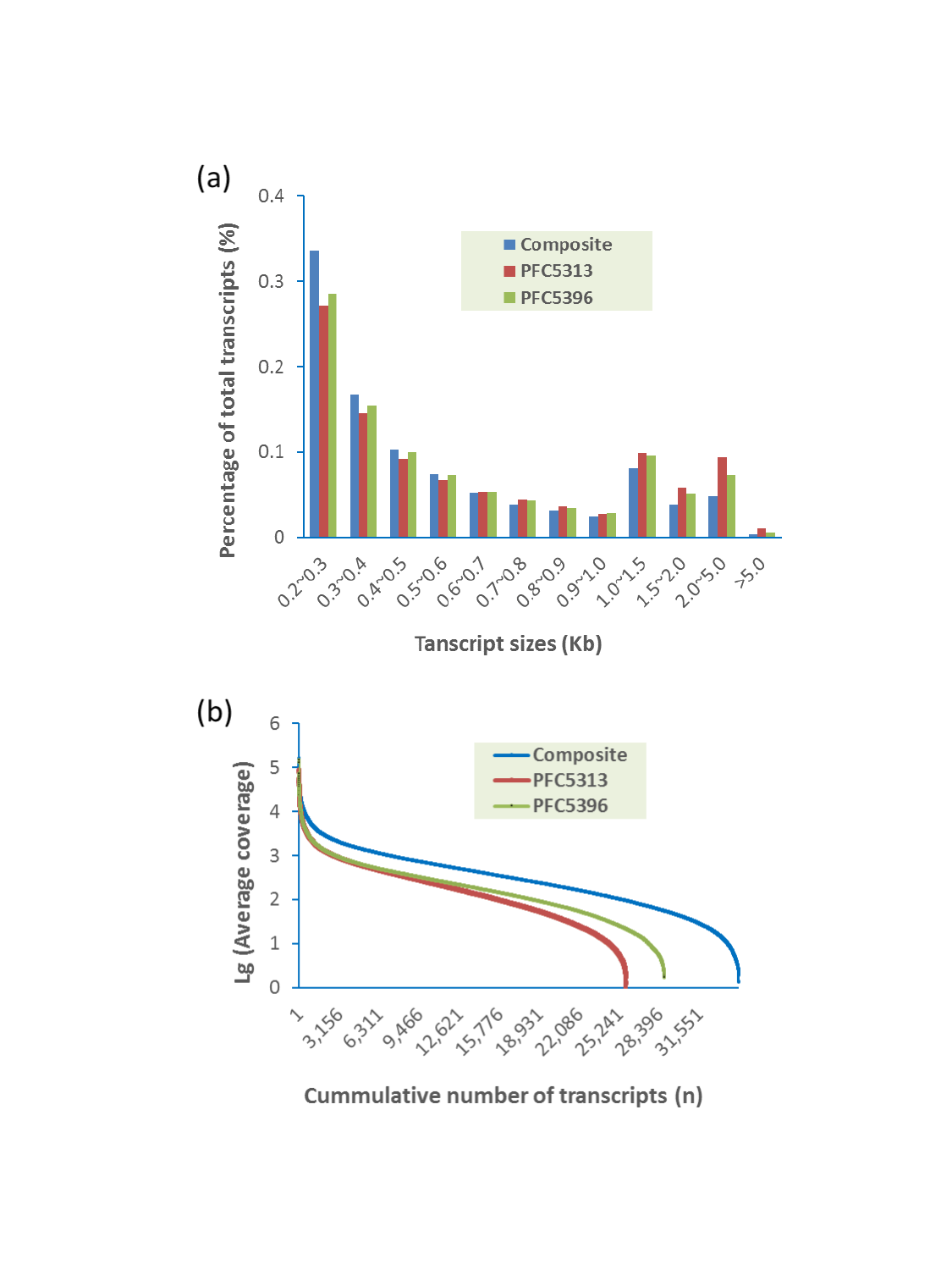

Supplement: Supplementary file 1 — Fig. S1. Transcriptomes de novo assembled by RNA‐seq analysis. [file MBT2-11-537-s001.pptx]

## Slide 1
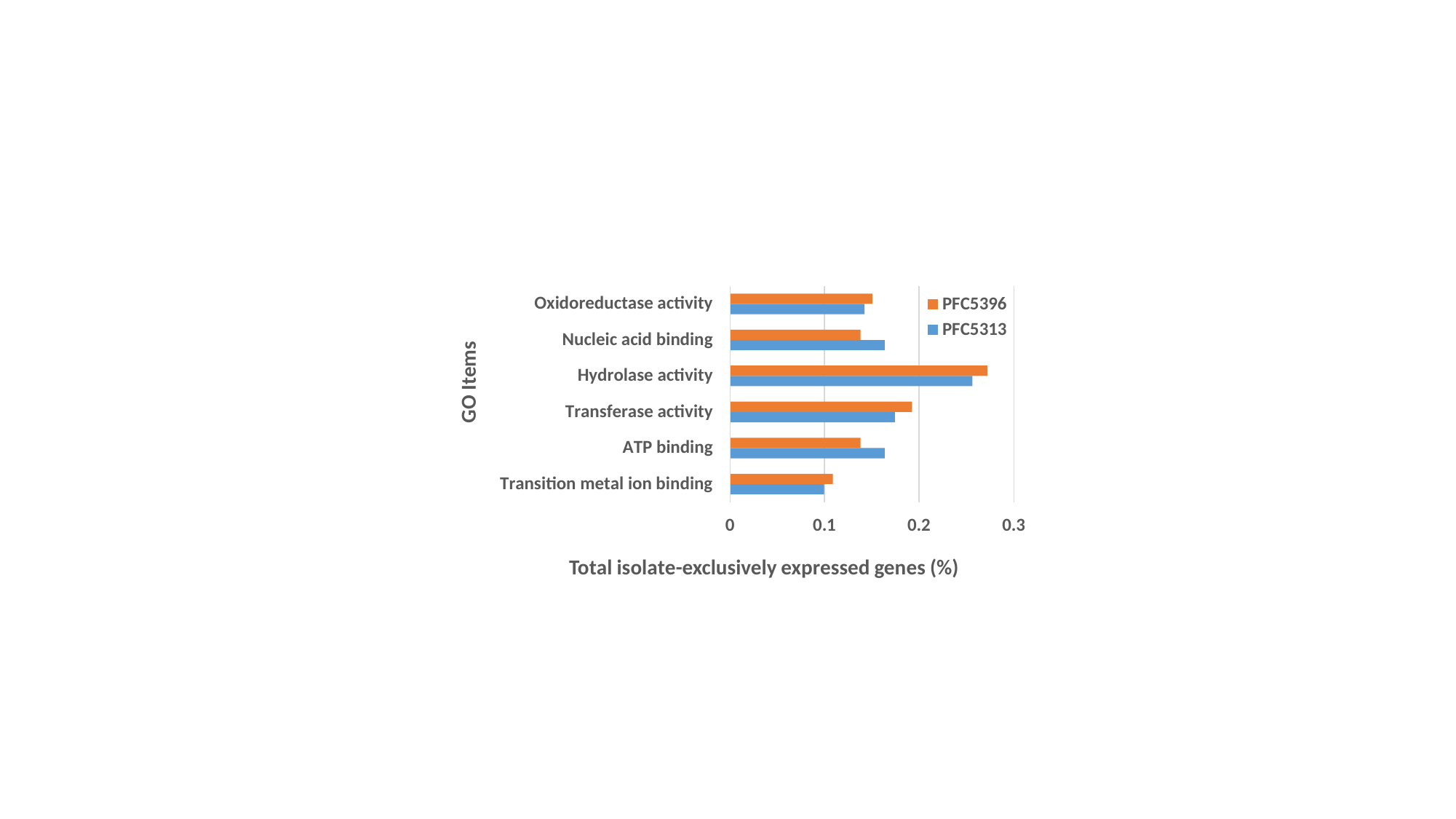

Supplement: Supplementary file 5 — Fig. S5. GO terms of H. occidentale genes with isolate‐exclusive expression patterns. [file MBT2-11-537-s005.pptx]

## Slide 1
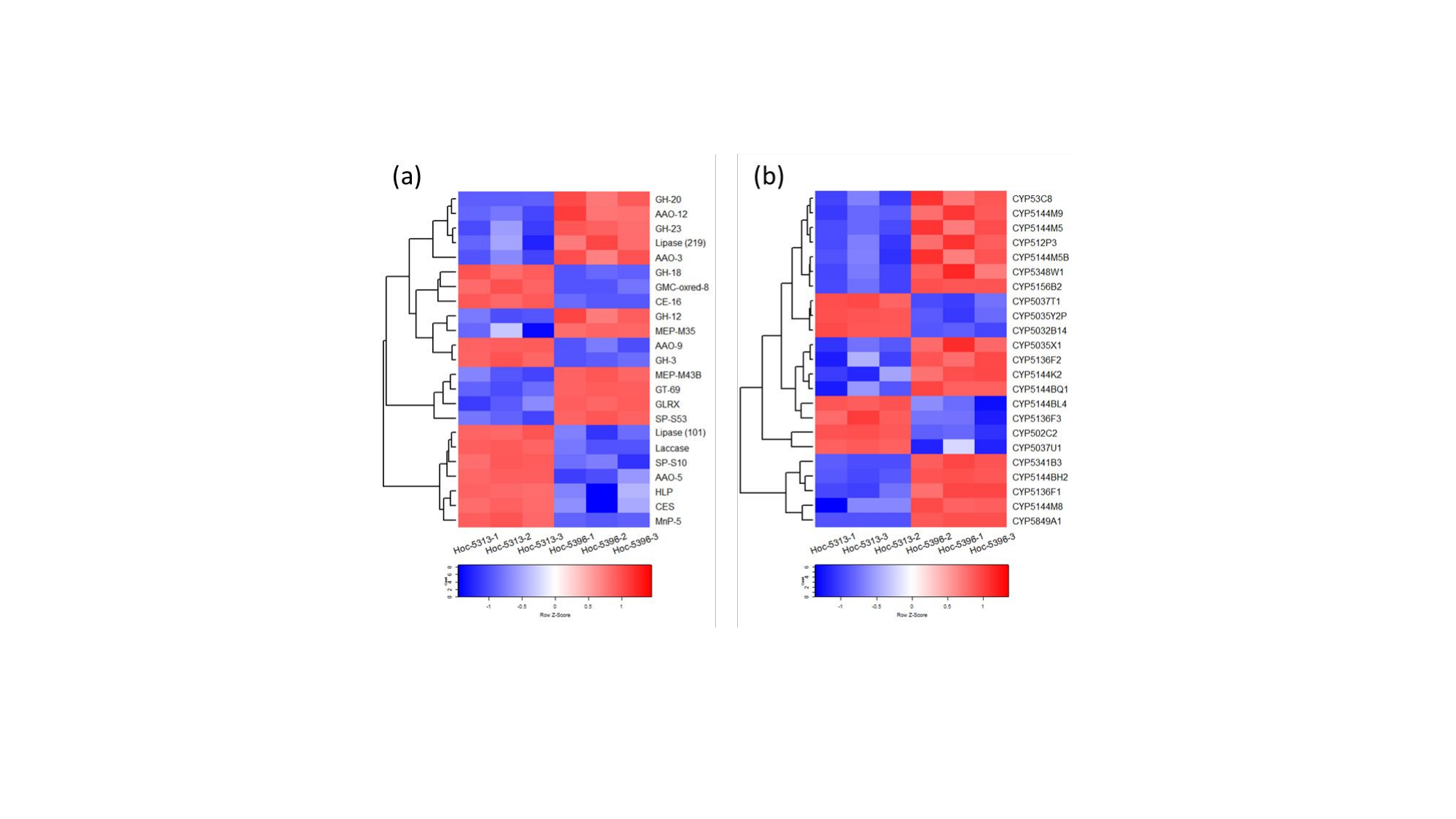

Supplement: Supplementary file 6 — Fig. S6. Heat maps showing the expression pattern of differentially expressed genes (DEGs) between the H. occidentale two isolates. [file MBT2-11-537-s006.pptx]
