# Supplementary material for: Characterization of Heterobasidion occidentale transcriptomes reveals candidate genes and DNA polymorphisms for virulence variations
Source: Microb Biotechnol. 2018 Apr 2;11(3):537–50. doi: 10.1111/1751-7915.13259 (PMC5954486; doi:10.1111/1751-7915.13259)
Supplement: Supplementary file 2 — Fig. S2. Protein sequence alignment of H. occidentale CYP5849A1 (Hoc‐contig15110) with its H. annosum ortholog (Han‐03520). [file MBT2-11-537-s002.docx]

Hoc-Contig15110 MSLTTYLPSDTTIALVVLLLTFSWIAFLLFYPLILPTNVPPLVDYTLPWLGHVFSLLSSP

Han_03520 MSLTTCLPSDTTIALVVLLLTFSWIAFLLFYPLILPTNAPPLVDYTLPWLGHVFSLLSSP

***** ********************************.*********************

Hoc-Contig15110 SDFMRECRSKHGTIYKIFARGQCIVVISDPCAIVSMQTKSPKVLGTMDFQKIHLLSGLTD

Han_03520 SDFMRECRSKHGPIYKIFARGQCIVVISDPCAIVSMQTKSPKVLGAMDFQKIHLLSGLTD

************ ********************************:**************

Hoc-Contig15110 RIPFVFEVLHRRVYSVATSSLTKRNLASVAIPINLQLFACLVGAVPDDMIVELDLQSFVG

Han_03520 RIPFIFEVLHRRVYSVATASLAKRNLASVAIPINLHLFACLESAVPNDMIVELDLQSFVG

****:*************:**:*************:***** .***:*************

Hoc-Contig15110 RSMYRACFSILFGPSFSLDTYSDYATFDNDMFYVMSGIPFTARNAKAARERLRSYMQSYL

Han_03520 RSTYRACFSILFGPSFSLDTYSDYATFDNDMFYVMSGIPFTARNAKAARERLRSYMQSYL

** *********************************************************

Hoc-Contig15110 EHNWRSDGGGHLDGASSVISCAVRELKDADLSDHEISCVLFIILWGIHSNMVQVTIWSIL

Han_03520 EHNWRSDGGGHMDGASSVISCAVRELKDADLSDHEISCVLFIILWGIHSNMVQVTIWSIL

***********:************************************************

Hoc-Contig15110 NLTNNSQTYDRIARDVRRAVERKASDFHSLLTADPSVLDDPDFAPLDSVVKETLRLSILP

Han_03520 NLTNNPQIYDRIARDVRRAVERKASDFHSLLTADPSVLDDPDFAPLDSVVKETLRLSILP

***** * ****************************************************

Hoc-Contig15110 STVRQVLHDTTIIGGNGKKYRIYKGEGVLVDVRGMHLDSDYFPDPESFKADRFMNIKGYG

Han_03520 STVRQVLHDTTIIGGNGKEYRIYKGEGVLVDVRGMHLDSDYFPDPESFKADRFMNIKGYG

******************:*****************************************

Hoc-Contig15110 EHNGMKTLVPWGGGMYMCKGRTFAQHMIKIFLIMCFHLYDINMHPNFSPPAASSAKSISV

Han_03520 EHNGMKTLVPWGGGMYMCKGRTFAQHMIKMFLIMCFHLYDIDMHPNSSPPAASSAKSISV

*****************************:***********:**** *************

Hoc-Contig15110 VRPDSALKIRLRKRRS

Han_03520 VRPDSALKIRLRKRQL

**************:

Sequence identity at 95.77%
