# Supplementary material for: Characterization of Heterobasidion occidentale transcriptomes reveals candidate genes and DNA polymorphisms for virulence variations
Source: Microb Biotechnol. 2018 Apr 2;11(3):537–50. doi: 10.1111/1751-7915.13259 (PMC5954486; doi:10.1111/1751-7915.13259)
Supplement: Supplementary file 3 — Fig. S3. Protein sequence alignment of H. occidentale CYP5348Z1 (Hoc‐contig733973) with its putative orthologs from Galerina marginata (KDR74420) and Hericium erinaceus (ARE72240). [file MBT2-11-537-s003.docx]

KDR74420 ------------------------------MVIFN--------FLASSWSIPPISTSAVV

ARE72240 ------------------------------------------MVLADFISIPTVSIAC--

Hoc-contig733973 MIRQVAAGSSAPKQKQMTFAWFSLISLSATMALVDSLVHSHLGRINVPPLIPAVGLAC--

: ** :. :.

KDR74420 GITLALVALKLFLHTSRRKRSFLPPGPPGYPFIGNLLQLAKAERPHLLFSGWNKQYGSIV

ARE72240 ---LAVLGIA-YHRHQSNKNTRRPPGPKGYPFIGNLLELVSAERPHLLFPLWIKQYGDIV

Hoc-contig733973 ---LVLLGLT-YRRRQAKQTLKRLPGPPGYPFIGNLLQLVGAERPHLLFPEWNRKYGDIV

*.::.: : : . .: *** *********:*. ******** * ::**.**

KDR74420 YFSVFGVPHIIINTFSAAHELLEKRGATYSDRPNMVLENDILGWDAAMPTMRYGAKFRKH

ARE72240 RFTVFGVENILISKFSTAIELLEKRGAIYSDRPHMVLENEILGWDAAMPTMRYGAQFRKH

Hoc-contig733973 QFSILGFQSIVINKFSIAVDLLEKRGAIYSDRPSMVLVTEILGWDTAMPVMRYGAQFRKH

*:::*. *:*..** * :******* ***** *** .:*****:***.*****:****

KDR74420 RRLSQALLNPTAASGYTRLHEDIAAELLSALMRRPEDFFDHILVYTVSILFFLTDLTSTF

ARE72240 RKLSNALLNPNAARGYIAIHEEVSLRLLSALAAQPDQFYHHILIY---------------

Hoc-contig733973 RRLSQALLNPNASRGYARLHEELANRLIAALTAHPEQFNDHTLIY---------------

*:**:*****.*: ** :**::: .*::** :*::* .* *:*

KDR74420 SYATSTIFRLAYDLDVRSEDHVLVKLANNAVRKSAEAYQASGSLVDFFPFLKPFYTSWPD

ARE72240 --ATSTIFRISYDIDITDDKHDLVRLANGAVRKSAEAYQASGALVDVFPSLKWLYNSYPT

Hoc-contig733973 --AASTIFKLTYDVDVTSEDHHLIRLANRAVKKSAEAFQASGAIVDVFPSLKWFYTSYPD

*:****:::**:*: .:.* *::*** **:*****:****::**.** ** :*.*:*

KDR74420 WAPLSGPKKTIAAIRDGVQKANNLPYDMAKEKMRTGDARPSLVQNAIKSFGGLESISSED

ARE72240 TAPFSGYRKVIEDIKGEVVQANNIPYEMAKEKMRDGSATRSLVSDAITGLGGLDAISPAD

Hoc-contig733973 KAPFSGFKTTAVDLRQEVDKANGIPYDMAKERI/NGTAQPSLVSDAINNSGGIQGISAED

**:** :.. :: * :**.:**:****:: * * ***.:**.. **::.** *

KDR74420 EHDIRGLAGILYGAGQETTMATLTTFILAMVRFPEAQKRARAEIDSVIPLDRLPNLDDRP

ARE72240 EHDIRGLAGILYA-GQETTMVTITNTILAMIHHPEVQRKAQAEIDAVVPLDRLPTLDDRA

Hoc-contig733973 EVDIRGLAGILYGAGQETTMATLNSFILAMIHNPEVQRKAQAAIDAVVPRGRLPNLNDRP

* **********. ******.*:.. ****:: **.*::*:* **:*:* .***.*:**

KDR74420 HLPYLEAFLKELYRFASPLIIAIPHTSIADDTYDGWVFPSGTTVIANICDMLNNCPRNEE

ARE72240 NIPYLEAFVKEAYRWACPLIIAIPHTVIQDDVYEGYFIPKGTSIIASIYDMLNQCPNAAA

Hoc-contig733973 DIPYLEAFIKELYRWASPLVIALPHAVIQDDFYGDYFIPKGTSIIASTIGMLNACPRPAD

.:******:** **:*.**:**:**: * ** * .:.:*.**::**. .*** **.

KDR74420 FLPHRFIDGTDLGDVPPDPRDVVFGFGRRRCPGMHVADNSLWTAVAGMLTMFEFSPEIVD

ARE72240 FNPDRFIDGTDLGDVPPDPRDVVFGFGRRRCPGLHVADNSVWAALAQLLASFEFLPELVD

Hoc-contig733973 FVPERFIDGTDLGDVPADPREVAFGFGRRRCPGIHVADNSIWTAVSQMLASFDFLPELVD

* *.************ ***:*.**********:******:*:*:: :*: *:* **:**

KDR74420 GKPVVPLQEFGIEMTRHPKLFRCCIQARADRRGLVTAWTN

ARE72240 GKEVLPPLKWGKEMARHPQPYRCRIVPRENRKHCYAA---

Hoc-contig733973 GKECLPPVKFGKEIIRHPEPFRCRIQPRDDHQRLVAV---

** :* ::* *: ***: :** * * ::: :.

# Percent Identity Matrix - created by Clustal2.1

#

#

1: KDR74420 100.00 63.92 61.94

2: ARE72240 63.92 100.00 66.67

3: Hoc-contig733973 61.94 66.67 100.00

ARE72240 [*Hericium erinaceus*]

KDR74420 [*Galerina marginata* CBS 339.88]
